# Molecular Characterization of *Cryptosporidium* spp. Isolated From Immunocompromised Patients and Children

**DOI:** 10.5812/jjm.9183

**Published:** 2014-04-01

**Authors:** Abdollah Rafiei, Zahra Rashno, Alireza Samarbafzadeh, Shahram Khademvatan

**Affiliations:** 1 Department of Medical Parasitology, School of Medicine, Ahvaz Jundishapur University of Medical Sciences, Ahvaz, IR Iran; 2 Infectious and Tropical Diseases Research Center, Ahvaz Jundishapur University of Medical Sciences, Ahvaz, IR Iran; 3 Department of Medical Virology, School of Medicine, Ahvaz Jundishapur University of Medical Sciences, Ahvaz, IR Iran

**Keywords:** *Cryptosporidium*, Epidemiology, Genotyping Techniques, RNA, Ribosomal, 18S, Iran

## Abstract

**Background:**

*Cryptosporidium* is known to be one of the most important causes of diarrhea in children and immunocompromised patients. Genotype characterization of *Cryptosporidium* species in each region would help in the treatment of this disease, as well as to locate the source of infection and to prevent the disease.

**Objectives:**

This current research was conducted in order to analyze the molecular characterization of isolated *Cryptosporidium spp.* in the Southwest of Iran.

**Materials and Methods:**

In this survey, 390 fecal samples were collected from immunocompromised individuals and children under five-years-of-age. Parasitic infection was evaluated using wet mount preparation, formalin ether, a modified acid fast staining method and microscopic examination. Finally, a PCR-RFLP assay was performed on the extracted DNA collected from fecal samples that were positive for *Cryptosporidium* by the acid fast method.

**Results:**

Among the 390 fecal samples, 16 cases (4.1%) were infected with *Cryptosporidium*. Molecular and genotype characterization found the following protozoan species; 11 *Cryptosporidium* *parvum *(68.8%), 4 *C.* *hominis* (25%), and one case of *C.* *meleagridis *(6.2%).

**Conclusions:**

The present study emphasized the public health importance of *Cryptosporidium spp.* in the study area. In addition, it seems that zoonotic species are the most important causes of infection in the region. As far as we are aware this the first report of a *C.* *meleagridis* infection in Iran.

## 1. Background

Cryptosporidiosis has been reported in both immunocompetent and immunocompromised patients and it has three major clinical presentations, including; asymptomatic carriage, acute diarrhea, and persistent diarrhea (1). *Cryptosporidium *spp. infection may cause severe clinical gastrointestinal disorders, especially in people infected with human immunodeficiency virus (HIV) (2), people with malignancies, patients with solid-organ transplants, and hemodialysis patients (3-6). Its prevalence has been reported to be 5-50% among patients with AIDS (7), and it is also an important cause of AIDS-associated deaths due to severe diarrhea. 

According to Plutzer and Karanis (8), 20 *Cryptosporidium* species have been recognized and approximately 61 *Cryptosporidium* genotypes have been found with uncertain species status, based on SSU-rRNA sequences. Five *Cryptosporidium* species/genotypes are responsible for the most common human cryptosporidiosis cases, including; *Cryptosporidium* *hominis*, *C. parvum, C. meleagridis*, *C. felis*, and *C. canis* (9). Among these species, *C.* *hominis* and *C. parvum* are the most common agents responsible for the majority of human infections, especially in industrialized nations, even though in some areas, the incidence of *C*. *meleagridis* infection is as high as *C. parvum* (10). A few other *Cryptosporidium* species and genotypes can occasionally cause human infections, including; *C. muris*, *C. suis*, *C. andersoni* and *C. cervine* and genotypes (9, 11, 12). 

The role of each transmission route in endemic areas remains unclear, and this is because of the ability of *Cryptosporidium* species to infect both humans and a wide variety of animals. In addition to the ubiquitous presence of the organism, the expensive nature of epidemiologic investigations, and the inability to differentiate *Cryptosporidium* species by conventional microscopic investigations, makes this process more difficult (13). Recently the use of molecular methods has significantly helped our understanding of the biology and epidemiology of *Cryptosporidium* species (14). This includes increased knowledge of the species structure and genetics in the *Cryptosporidium* population, the roles of various transmission routes in cryptosporidiosis epidemiology, and the significance of parasite genetics in pathogenesis and clinical presentations (15). These recent developments have enabled researchers to provide more accurate risk assessments on environmental and drinking water contamination, and it has also helped health officials to better educate the public about the risk factors involved in the acquisition of cryptosporidiosis in vulnerable populations (16, 17). There is little information regarding cryptosporidiosis in the region and no current molecular *Cryptosporidium* characterization data are available.

## 2. Objectives

The current study was conducted to determine *Cryptosporidium* genotypes isolated from immunocompromised patients in the Southwest of Iran. 

## 3. Materials and Methods

### 3.1. Sample Collection

In this study, 390 stool samples were collected from different groups, including; HIV infected patients, patients with hematological malignancies undergoing chemotherapy, kidney transplant recipients, and children younger than five-years-of-age. A smear was stained using a modified Ziehl-Neelsen method for the detection of *Cryptosporidium* spp. The positive samples were preserved in 2.5% potassium dichromate (K2Cr2O7) and stored at 4˚C for DNA extraction. 

### 3.2. DNA Extraction

*Cryptosporidium* oocysts were purified using a sucrose flotation procedure (18). Purified oocysts were washed three times in PBS. Samples were centrifuged at l000×g for 20 minutes. The pellets were then subjected to 5 - 8 freeze-thaw cycles and DNA extraction was carried out using a QIAamp DNA Stool Mini Kit (QIAamp® DNA Stool Mini Kit, USA). Briefly, the total pellet resulting from the sucrose flotation was dissolved in 2 mL ASL buffer. A total of 1.4 mL of stool lysate was transferred to a 2 mL centrifuge tube and incubated at 70°C for 15 minutes. All procedures were carried out using a QIAamp DNA Stool Mini Kit according to the manufacturer’s instructions.

### 3.3. Cryptosporidium Genotyping

*Cryptosporidium* oocysts were identified using a small-subunit rRNA-based on nested PCR, as described by Xiao et al. (19). Primary PCR was performed by primers SSU-F2: (5 TTCTAGAGCTAATACATGCG 3) and SSU-R2: (5 CCCATTTCCTTCGAAACAGGA 3) (19-22). The primary PCR mixtures contained 10 µL of template, 10X PCR buffer, 10 mM deoxynucleoside triphosphate mix (dNTP), 3 mM MgCL_2_, 50 pmol of each primer, and 0.3 U of Taq DNA polymerase in a 50 µL reaction volume. The secondary PCR was performed using the following primers SSU-F3: (5 GGAAGGGTTGTATTTATTAGATAAAG 3) and SSU-R4: (5 CTCATAAGGTGCTGAAGGAGTA 3) (21). 

The reaction conditions were similar to those described above in the primary PCR, except that 5 μL product of the previous PCR was used as the template. Thermocycling parameters were 4 minutes at 94°C hot start (initial heat activation step), followed by 35 cycles of 45 seconds at 94°C, 90 seconds at 58°C and 1 minute at 72°C, with a final extension of 7 minutes at 72°C (19). The PCR product was loaded on 1% agarose gel, electrophoresed for 1 hour. The gel was stained with ethidium bromide and the products were visualized under a UV transilluminator (Uvitec, UK). The size of the amplicon was approximately 830 bp. 

### 3.4. RFLP (Restriction Fragment Lngth Polymorphism)

Restriction assays were conducted in a 30 μL volume with 0.5 units of restriction enzymes and 20 μL of PCR product per reaction. Mixes were incubated at 37°C for 8 hours. Digested products were visualized under UV light after 1% agarose gel electrophoresis and ethidium bromide staining. The endonuclease enzymes used were* SspI* and *VspI* (Vivantis, Malaysia). 

## 4. Results

**Figure 1.**
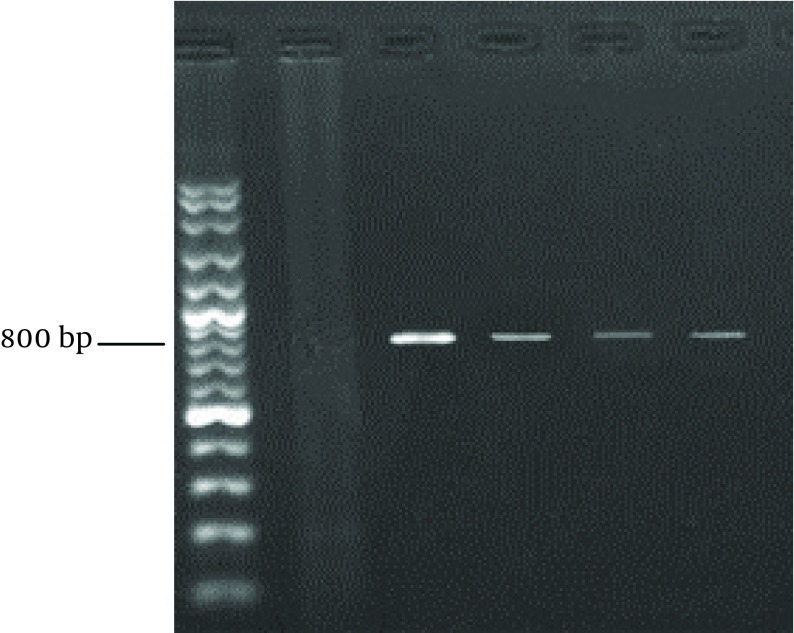
Nested PCR Products for *Cryptosporidium* spp. SSUrRNA gene sequences from three patients (lanes 4-6); Lane 1=100 bp marker; Lane 2 = negative control; Lane 3 = positive control for *Cryptosporidium*.

**Figure 2.**
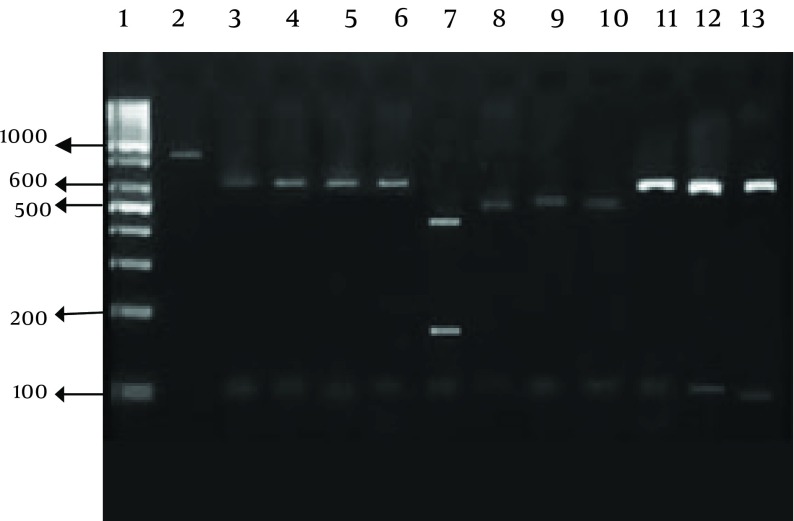
PCR-RFLP of *Cryptosporidium* SSUrRNA Gene Secondary PCR product was digested by *VSPI* restriction enzymes. Lane 1, DNA size marker. Lane 2, undigested PCR product. Lanes 3-6 and 11-13, *C. parvum* 628,104 bp. Lane 7, C. *meleagridis* 457 171 and 115 bp. Lanes 8-10, *C. hominis* 556 and 116 bp.

Our research showed an incidence of 4.1% (16/390) *Cryptosporidium* infection among immunocompromised patients using a microscopic. Higher rates of infection were identified among HIV+ and kidney transplant recipients (Table 1 and Figure 1). RFLP analysis of the PCR products revealed the presence of three *Cryptosporidium* species in human cases in the Southwest of Iran. The isolates were comprised of; 11 cases of *C. parvum*, four *C. hominis*, and one *C. meleagridis* case (Table 2 and Figure 2). 

**Table 1.** Frequency of *Cryptosporidium* Species Among Immunocompromised Patients and Children, Southwest of Iran

Cases	Tested Cases, No. (%)	Infected Cases, No. (%)	*Cryptosporidium *Species
**Malignant hematologic children**	132 (33.8%)	4 (3)	*C. hominis*, *C. parvum*
**Malignant hematologic adults**	111 (28.5)	4 (3.6)	*C. hominis*, *C. parvum*
**Kidney transplant recipients**	48 (12.3)	3 (6.2)	*C. hominis*, *C. parvum*
**HIV+**	48 (12.3)	3 (6.2)	*C. parvum*
**Hemodialysis patients**	32 (8.2)	1 (3.1)	*C. parvum*
**Children younger than 5 years**	19 (4.9)	1 (5.2)	*C. meleagridis*

**Table 2.** *Cryptosporidium* Species Isolated from Immunocompromised Patients and Children, in the Southwest of Iran

*Cryptosporidium* Species	Patients, No. (%)
***C.parvum***	11 (68.8)
***C.hominis***	4 (25)
***C.meleagridis***	1 (6.2)

## 5. Discussion

*Cryptosporidium* can cause a self-limiting infection in immunocompetent cases; on the other hand, it can lead to severe illness in immunocompromised patients. Molecular tools have been developed to detect and differentiate *Cryptosporidium* species/genotype and its subtype levels (23, 24). Our study focused on the current state of *Cryptosporidium* infection in immunocompromised patients and children younger than five-years-of-age referring to hospitals in Ahvaz City, Southwest of Iran.

In the present study, the prevalence of Cryptosporidium was 4.1% (16/390). A previous study in this region reported a prevalence of 5.1% (9/179) among immunocompromised patients, which is similar to our report (25). It seems that Cryptosporidium infection is still one of the opportunistic parasitic infections in this region that can result in life threatening disease, especially among immunocompromised patients. After molecular analysis, three different Cryptosporidium species were detected including *C. parvum*, C. hominis and *C. meleagridis*. This is the first molecular characterization of *Cryptosporidium* collected from human patients in the Southwest of Iran. Of the 16 isolates, 68.75% were *C. parvum*, 25% C. hominis and 6.25% C. *meleagridis*. The predominance of *C. parvum* may be due to the high prevalence of bovine cryptosporidiosis in this region. The results were in close agreement with previous studies conducted in Iran (26, 27). 

Molecular characterization of *Cryptosporidium* isolates from humans and animals in Iran showed a frequency of 74% *C. parvum* and 26% *C.* *hominis* among infected cases in some parts of Iran (26). Additional research in Shahriar (suburb of Tehran) identified 18 out of 24 (75%) isolates from patients with diarrhea as *C. parvum* (27). These reports were in close agreement with our results. In contrast, in another study *C. hominis* (71%) was the most predominant species found among Iranian patients infected with HIV (28). In Saudi Arabia, the incidence of *C. parvum* was 42.9%, followed by 37% of C. *hominis*, C. *meleagridis* and *C. muris* in one sample (29). In Kuwait *C. parvum *and *C.* *hominis* were detected in 73% and 27% of isolates, respectively (30). 

We identified one patient infected with *C.* *meleagridis*. As far as we are aware this is the first report of a *C.* *meleagridis* infection in human hosts in Iran. This species has formerly been reported in other locations, such as; Portugal (31), India (24, 32), Taiwan (33), and the UK (33). 

In conclusion, *C.* *parvum* and *C*. *hominis* were the dominant species isolated from immunocompromised patients in the current study. Furthermore, a high incidence of the predominant zoonotic infection in our study revealed that water resource contamination with animal offal may be the main source of infections in the region; therefore, further studies in different environmental areas are needed in order to better understand the route of *Cryptosporidium* transmission.
